# Tetrameric Neuraminidase of Influenza A Virus Is Required to Induce Protective Antibody Responses in Mice

**DOI:** 10.3389/fmicb.2021.729914

**Published:** 2021-10-04

**Authors:** Xiren Deng, Qimin Wang, Mei Liu, Qinwen Zheng, Fan Wu, Jinghe Huang

**Affiliations:** Shanghai Public Health Clinical Center and Key Laboratory of Medical Molecular Virology (MOE/NHC/CAMS), School of Basic Medical Sciences, Shanghai Medical College, Fudan University, Shanghai, China

**Keywords:** influenza virus, neuraminidase, protein, vaccine, cross-protection

## Abstract

Influenza neuraminidase (NA) is able to induce cross-subtype immunity and is considered as a promising target for the development of universal influenza vaccines. However, commercial influenza vaccines only induced low NA-specific immune responses due to the low amounts and the denatured conformation of NA proteins in current inactivated or split influenza vaccines. Here we investigated the protective efficacy of recombinant tetrameric and monomeric NA proteins to determine whether the conformation contributed to induce protective immunity. We found that H1N1_*P*__*R*__8_NA tetramer (NA_*tet*_) could provide complete homologous protection against A/PR8 (H1N1) virus infection in mice, while the protection of H1N1_*P*__*R*__8_NA monomer (NA_*mono*_) was moderate. Higher levels of NA-reactive binding and inhibition antibodies and less weight loss were observed in the H1N1_*P*__*R*__8_NA_*tet*_-vaccinated group. Similarly, H5N1_*V*__*N*_NA_*tet*_ immunization exhibited a preferable heterologous protection than H5N1_*V*__*N*_NA_*mono*_, but neither H7N9_*S*__*H*_NA_*tet*_ nor H7N9_*S*__*H*_NA_*mono*_ vaccination showed heterosubtypic protection. We also compared the effect of three adjuvants, aluminum, 3′3′-cGAMP (cGAMP), and Poly(I:C), on the humoral response and protective efficacy induced by H1N1_*P*__*R*__8_NA_*tet*_. H1N1_*P*__*R*__8_NA_*tet*_ protein adjuvanted with aluminum was observed to exhibited better capacity in inducing NA-specific humoral immunity and preventing weight loss than with cGAMP or Poly(I:C). In conclusion, our data demonstrate that tetrameric NA with natural conformation is required to induce protective anti-NA immunity. The NA tetramer could provide homologous protection and subtype-specific cross-protection. In addition, the aluminum adjuvant is preferable in recombinant NA protein vaccination.

## Introduction

The seasonal epidemics and less frequently global pandemics of influenza viruses result in high morbidity and mortality every year. Vaccination is the most effective way to prevent influenza circulation. The currently licensed vaccines, including inactivated and attenuated influenza vaccines, induce protection mainly by eliciting neutralizing antibodies (NAbs) against the major glycoprotein, hemagglutinin (HA), of influenza virus (Vogel and Manicassamy, 2020). Vaccine efficacy depends on the antigenic similarities of HA proteins between the vaccine and circulating strains. However, the continuous and extensive antigenic variation of HA protein allows influenza viruses to easily escape from the protection of vaccines. The vaccine components need to be updated yearly based on the prediction of circulating influenza strains. The mismatch of vaccine and circulating strains, as in the 2009–2010 and 2017–2018 influenza seasons, resulted in a significant increase of influenza-related morbidity and mortality. A universal vaccine may overcome the shortage of the current influenza vaccines and provide cross-protection against multiple influenza subtypes.

Neuraminidase (NA) protein is the second most abundant glycoprotein on the membrane of influenza virion. The native influenza NA protein is a tetramer with enzymatic activity (Saito et al., 1995; Wu et al., 2009; Da Silva et al., 2013) that can cleave off the terminal sialic acid from N-linked glycans to facilitate viral release and transmission (Krammer et al., 2018; Eichelberger and Monto, 2019). Serologic studies showed that individuals with higher NA-specific antibody titers were less likely to be infected by influenza virus with the same NA subtypes (Murphy et al., 1972; Monto and Kendal, 1973). NA inhibition (NAI) antibody titers were independently correlated with lower morbidity and decreased viral shedding in influenza-infected individuals (Couch et al., 2013; Stadlbauer et al., 2019). Although there are 11 different subtypes of NA proteins, N10 and N11 NA are unique to bats (Tong et al., 2012, 2013). The antigenic variation of NA protein is relatively low compared to that of HA protein (Couch et al., 2013; Stadlbauer et al., 2019). Furthermore, NA-specific monoclonal antibodies isolated from influenza-infected individuals provide cross-protection against multiple influenza virus strains (Chen et al., 2018; Stadlbauer et al., 2019; Yasuhara et al., 2019). Therefore, NA protein is considered a candidate for the development of universal influenza vaccines. Recent studies have shown that expressed or purified NA proteins could induce robust NA-based immunity and protect against influenza virus infection (Liu et al., 2015; Wohlbold et al., 2015; McMahon et al., 2020), indicating that the NA protein is immunogenic in both animal models and humans. In contrast, commercial inactivated and split influenza vaccines only induced low levels of NA-specific immune response (Wohlbold et al., 2015; Krammer et al., 2018). Several factors were suggested to contribute to the low immunogenicity of NA in inactivated and split influenza vaccines. First, the amount of NA proteins is relatively lower than HA in the vaccine formula (Sridhar et al., 2015; Wohlbold et al., 2015); second, NA seems to have immune subdominance to HA when both antigens were administered (Johansson et al., 1987; Krammer et al., 2018); and third, the NA conformation in the vaccine formula may be changed during vaccine manufacturing (McMahon et al., 2020).

In this paper, we investigated the factors that potentially affect the immune response and protective efficacy induced by NA protein in BALB/c mouse models. We compared the difference of the protective efficacy between tetrameric and monomeric NA proteins and investigated the humoral responses induced by tetrameric and monomeric NA proteins. We also assessed the influence of adjuvants on the humoral response and protective efficacy induced by H1N1_*P*__*R*__8_NA_*tet*_ tetramer.

## Materials and Methods

### Cells and Viruses

Madin Darby canine kidney (MDCK) and human embryonic kidney 293 (HEK293T) cells were obtained from the American Type Culture Collection and grown in complete high-glucose Dulbecco’s modified Eagle’s medium (DMEM, HyClone) supplemented with antibiotics (100 units/ml penicillin and 100 μg/ml streptomycin, HyClone) and 10% fetal bovine serum (FBS, Gibco). Expi293F (Thermo Fisher Scientific) cells were grown in SMM 293-TII expression medium (Sino Biological Inc.).

The influenza A virus (IAV) used in this study was the mouse-adapted strain A/Puerto Rico/8/1934 (H1N1). The IAV was propagated in MDCK cells in serum-free DMEM media in the presence of 1 μg/ml TPCK-trypsin (Sigma-Aldrich). The median tissue culture infective dose and median lethal dose (LD_50_) of viruses were calculated by the Reed and Munch method.

### Expression and Purification of Recombinant Proteins

Recombinant NA proteins derived from A/Puerto Rico/8/1934 (H1N1), A/Shanghai/37T/2009 (H1N1), A/Hong Kong/16/68 (H3N2), A/Vietnam/1204 (H5N1), and A/Shanghai/4664T/2013 (H7N9)—referred to as H1N1_*P*__*R*__8_NA, H1N1_*p*__0__9_NA, H3N2_*H*__*K*_NA, H5N1_*V*__*N*_NA, and H7N9_*S*__*H*_NA, respectively—were expressed in Expi293F cells and purified by Ni-nitrilotriacetic acid (NTA) beads (GE Healthcare). Briefly, the NA ectodomains with additional N-terminal Igκ-light chain secretion sequence, followed by a hex-histidine tag (HHHHHH), a human vasodilator stimulating phosphoprotein (VASP) tetramerization domain (SSSDYSDLQRVKQELLEEVKKELQKVKEEIIEAFVQELRKRG), and a thrombin cleavage site (SLVPRGSPSRS) were constructed into eukaryotic expressing plasmid pcDNA3.1 to express NA tetramer (NA_*tet*_). The monomeric NA (NA_*mono*_) proteins were constructed in the same way as NA_*tet*_ but without the VASP domain. Recombinant NA proteins were purified from the supernatant of transiently transfected Expi293F cells by Ni-NTA beads. The concentration of proteins was adjusted to 1 mg/ml with phosphate-buffered saline (PBS) and frozen at −80°C.

### Western Blotting and Cross-Linking SDS-PAGE

Recombinant NA proteins were then analyzed by Western blotting. Briefly, 2 μg of NA were mixed with 4× SDS-loading buffer containing 10% β-mercaptoethanol. The samples were heated for 10 min at 98°C and were afterward loaded on an SDS gradient gel (4–20% Precast Protein Improve Gels, Yeasen Biotechnology Inc.). The gel was run for 100 min at 120 V, and Western blotting transfer was performed. Following the transfer onto the nitrocellulose membrane, the membrane was blocked with TBS with 0.1% Tween 20 (TBS-T) containing 5% milk powder for 2 h at room temperature (RT). Then, mouse anti-HIS primary antibody (Younuoke Biotechnology Inc.) was added for 12 h at 4°C (1:1,000 dilution in TBS-T containing 1% milk). The membrane was washed three times with TBS-T after 12 h, and a secondary goat anti-mouse horse radish peroxidase (HRP) antibody (Jackson Immuno Research) was added for 1 h at RT (diluted 1:1,000 in PBS-T containing 1% milk). The membrane was then washed three times with TBS-T before it was visualized using Pierce Chemiluminescence (ECL) Western Blotting Substrate (as per the instructions of the manufacturer) on Tanon-5200 Chemiluminescent Imaging System (Tanon Science and Technology).

The extent of tetramerization and/or multimerization was investigated by cross-linking of NA with glutaraldehyde (Sigma-Aldrich). Briefly, 5 μg of NA was diluted in 25 μl of PBS in the presence of 0.3 mM of glutaraldehyde cross-linker. The mixture was incubated at RT for 5 min, and then glutaraldehyde was quenched by adding 1 M Tris–HCl buffer (pH 8.0) to a final concentration of 50 mM. Afterward, the protein samples were loaded on 4–20% SDS gradient gel. The gel was run for 60 min at 180 V and confirmed by Coomassie staining.

### Neuraminidase Enzymatic Activity Assay

The enzymatic activity of recombinant NA proteins was determined by the cleavage of two specific substrates of NA, 2′-(4-methylumbelliferyl)-α-D-N-acetylneuraminic acid (MUNANA) (Job et al., 2018; Ju et al., 2018) and fetuin (Prevato et al., 2015; Biuso et al., 2019) as previously described, with minor modifications.

For the MUNANA-based enzymatic activity assay, twofold gradient diluted recombinant NA proteins that range from 0.0015625 to 1.6 μg/ml in morpholine ethanesulfonic acid (MES) buffer (32.5 mM MES and 2 mM CaCl_2_, pH 6.5) were incubated with 20 mM MUNANA (Sigma-Aldrich) at 37°C for 40 min. The reaction was stopped by adding a stopping buffer (0.2 M glycine and 0.2 M NaOH, pH 10.7), and the degree of fluorescence was detected by EnSight Multimode plate reader (PerkinElmer).

For the fetuin-based enzyme-linked lectin assay (ELLA), MaxiSorp Nunc-immuno 96-well plates were coated with 100 μl of fetuin (Sigma-Aldrich) at a concentration of 50 μg/ml and refrigerated at 4°C overnight. The plates were blocked with 200 μl blocking buffer (PBS containing 1% FBS and 5% dry milk) for 1 h at RT and washed six times with PBS-T solution. Following the blocking, 50 μl of the sample diluent [Dulbecco’s phosphate buffered saline (DPBS) with 1% bovine serum albumin and 0.5% Tween 20] and 50 μl of serially diluted recombinant NA protein or purified virus were then added to the fetuin-coated plates and mixed well. The plates were then incubated for 14–16 h at 37°C before being washed six times with PBS-T. After the extensive wash, the NA enzymatic activity was detected by horse-radish peroxidase-labeled peanut agglutinin (Sigma Aldrich) and developed with the 3,3′,5,5′-tetramethylbenzidine substrate (Sigma-Aldrich). The reaction was stopped by the addition of 2 N H_2_SO_4_ after 20 min of incubation. The optical density (OD) values were read at 450 nm on a Multiskan FC plate reader (Thermo Fisher Scientific).

### Mice Experiments

Female BALB/c mice (6–8 weeks old) from the Laboratory Animal Center of Shanghai Public Health Clinical Center (SHPHCC) were used for all animal experiments. The protocols were reviewed and approved by the Ethics Committee of SHPHCC (approval no. 2019-A019-01/02).

To compare the immunogenicity and protective efficacy of tetrameric and monomeric NA proteins, the mice were intraperitoneally (i.p.) immunized with 20 μg H1N1_*P*__*R*__8_NA_*tet*_, H5N1_*V*__*N*_NA_*tet*_, H7N9_*S*__*H*_NA_*tet*_, H1N1_*P*__*R*__8_NA_*mono*_, H5N1_*V*__*N*_NA_*mono*_, and H7N9_*S*__*H*_NA_*mono*_, respectively. The antigens were diluted in 100 μl PBS and mixed with aluminum adjuvant (1:1). The mice were immunized with PBS as control. To investigate the influence of adjuvants on the immunogenicity of NA tetramers, the mice were intraperitoneally immunized with 20 μg H1N1_*P*__*R*__8_NA_*tet*_ protein mixed with aluminum (i.p.), 3′3′-cGAMP (cGAMP; intradermally, i.d.) and Poly(I:C) (i.p.), respectively. Aluminum is a strong inducer of Th2 responses (Rudicell et al., 2019). cGAMP is a Th1 immune response inducer as an “ideal” adjuvant for cutaneous vaccination (Wang et al., 2016). The TLR3 agonist Poly(I:C) also promotes Th1-dominant immunity, and it is commonly used as intraperitoneal immune adjuvant (Moriyama et al., 2017). These three adjuvants were all purchased from InvivoGen. The mice were boosted with the same immunogen 2 weeks later.

Sera were collected 1 week after the final immunization to detect NA-specific antibodies. The mice were intranasally challenged with 5 LD_50_ of A/Puerto Rico/8/34 (H1N1) virus 2 weeks after the final immunization to evaluate the protective efficacy. The weight loss and survival rates of mice were monitored for 14 days after the challenge.

### Enzyme-Linked Immunosorbent Assay

The NA-specific antibodies were measured by enzyme-linked immunosorbent assay (ELISA). To explore the immunogenicity of tetrameric and monomeric NA proteins, the sera samples which were collected 1 week after the final immunization were tested for NA-reactive antibodies by ELISA, including NA-specific binding antibodies to the respective immunogen, cross-subtype binding antibodies against NA of A/PR8 (H1N1), and NAI antibodies. The subtypes of NA-specific antibodies were also analyzed. Briefly, MaxiSorp Nunc-immuno 96-well plates (Thermo Fisher Scientific) were coated with 2 μg/ml NA proteins (100 μl/well) in carb/bicarb coating buffer (10 mM Na_2_CO_3_, 40 mM NaHCO_3_, and pH 9.6) at 4°C overnight. To detect the NA-specific antibodies, the respective immunogen to sera of immunized mice was coated. To detect the cross-binding activity of mice sera, the tetrameric NA protein of H1N1_*P*__*R*__8_NA_*tet*_ was coated. The plates were washed three times with PBS-T (PBS containing 0.05% Tween 20) and blocked with blocking buffer (PBS containing 1% FBS and 5% dry milk) for 1 h at RT. The sera were fourfold serially diluted starting at 1:100 in disruption buffer (PBS containing 5% FBS, 2% BSA, and 1% Triton X-100). Then, 50 μl of diluted serum was added to each well and incubated for 1 h at RT. After three times of washing with PBS-T, bound antibodies were detected by horseradish peroxidase-labeled goat-anti mouse IgG antibody (Jackson Immuno Research) and substrate ABTS (Thermo Fisher Scientific). The OD was measured at 405 nm on a Multiskan FC plate reader (Thermo Fisher Scientific). The isotypes of NA-specific antibodies were determined by ELISA with Mouse Monoclonal Antibody Isotyping Reagents (Sigma-Aldrich) following the instructions of the manufacturer. The antibody titers were defined as the highest sera dilution at which the OD values were twice of those by the control sera.

### Neuraminidase Inhibition Assay

The plates were coated and blocked as fetuin-based ELLA as described above. While the plates were blocked, pre-challenge mouse sera were fourfold serially diluted starting at 1:40 in PBS in a new U-bottom, 96-well plate. Then, the split A/PR8 (H1N1) virus was added to each well of the serially diluted serum plate, and the plates were incubated at 37°C for 1 h. The amount of split A/PR8 (H1N1) virus used in the NAI assay corresponded to 90% of the maximum signal. After incubation, 100 μl of the serum/virus mixture was added on the blocked fetuin-coated plates and incubated for 16–18 h at 37°C. The remainder of the NAI assay was performed as ELLA as described above. The values of test wells obtained from the plate reader were divided by the average value for virus-only control wells and then multiplied by a factor of 100 to obtain the NA activity. Percent inhibition was calculated by subtracting the NA activity from 100.

### Statistical Analysis

The survival rate was compared by log-rank test. Antibody titers, viral titers, and body weights among groups were compared by one- or two-way ANOVA. A *P*-value < 0.05 was considered significant. All the statistical analyses were performed by GraphPad Prism, version 8.00 (GraphPad Software, San Diego, CA, United States).

## Results

### Neuraminidase Tetramers, but Not Monomers, Exhibited Enzymatic Activity

**FIGURE 1:**
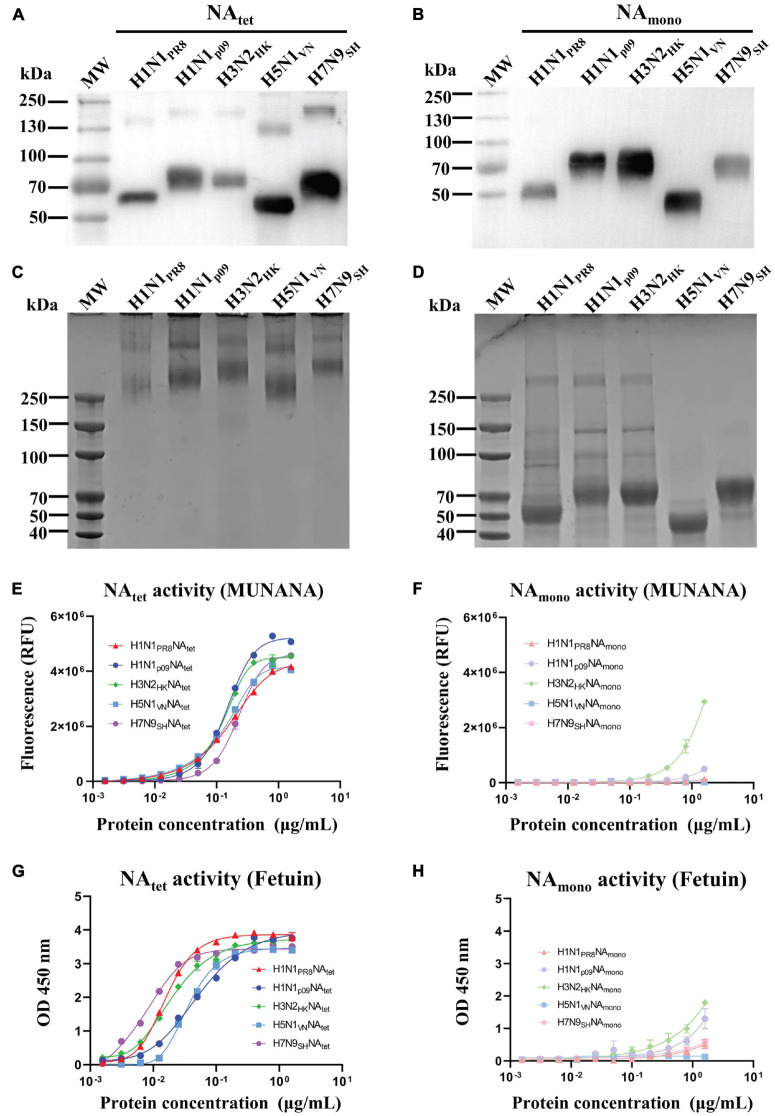
Expression, purification, and characterization of recombinant neuraminidase (NA) proteins. Tetrameric and monomeric NA proteins derived from A/Puerto Rico/8/1934 (H1N1), A/Shanghai/37T/2009 (H1N1), A/Hong Kong/16/68 (H3N2), A/Vietnam/1204 (H5N1), and A/Shanghai/4664T/2013 (H7N9) were expressed by the Expi293F cell expression system and purified by Ni-nitrilotriacetic acid (NTA). **(A,B)** Purified NA proteins were confirmed by Western blotting. **(C,D)** Cross-linking SDS-PAGE of tetrameric and monomeric recombinant NA proteins. **(E,F)** MUNANA-based enzymatic activity assays were used to determine the enzymatic activity of recombinant NA proteins. **(G,H)** Fetuin-based enzymatic activity assays were used to determine the enzymatic activity of recombinant NA proteins.

The NA tetramers and monomers were expressed using Expi293F cells and purified by Ni-NTA. The proteins were analyzed by Western blotting (Figures 1A,B) and cross-linking SDS-PAGE (Figures 1C,D). The bands of NA tetramers stabilized by the VASP domain were observed as monomers on Western blotting (Figure 1A), while they showed bands consistent with tetramers when cross-linked (Figure 1C). The monomeric NA proteins exhibited as monomers on Western blotting (Figure 1B) and showed some level on dimer or tetramer formation when cross-linked but was mostly monomeric (Figure 1D). The enzymatic activity of NA tetramers and monomers was characterized using two NA-specific substrates, MU-NANA and fetuin, respectively. These assays measured the amount of 4-methylumbelliferone cleaved by the influenza virus NA from the MUNANA or the amount of NA cleaved-fetuin from the intact fetuin. For the results of MUNANA-based enzymatic activity assays, as shown in Figures 1E,F, the NA tetramers exhibited enzymatic activity, while the NA monomers did not. The same results were also observed in fetuin-based enzymatic activity assays; the tetrameric NA showed high enzymatic activity, but the monomeric NA did not (Figures 1G,H). Taken together, these results suggest that the enzymatic activity depends on the tetrameric conformation of the NA proteins.

### Tetrameric Neuraminidase Protein Induced Better Protection Against Influenza Challenge in Mice Than Monomeric Neuraminidase

**FIGURE 2:**
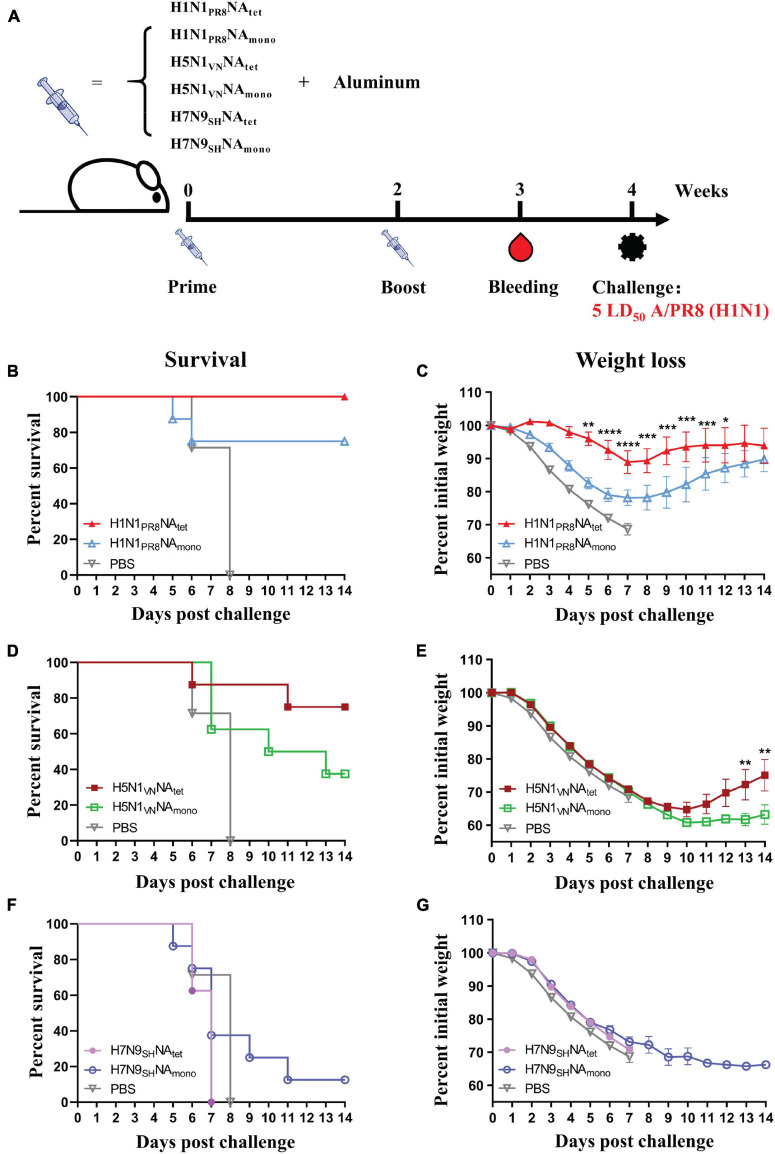
Protective efficacy of tetrameric neuraminidase (NA) vs. monomeric NA. **(A)** The experimental design for immunization and challenge studies. Six- to eight-week-old BALB/c mice (*n* = 8 in each group) were immunized twice at 2-week interval with 20 μg of H1N1_*P*__*R*__8_NA_*tet*_, H5N1_*V*__*N*_NA_*tet*_, H7N9_*S*__*H*_NA_*tet*_, H1N1_*P*__*R*__8_NA_*mono*_, H5N1_*V*__*N*_NA_*mono*_, and H7N9_*S*__*H*_NA_*mono*_ proteins adjuvanted with aluminum (i.p.), respectively. Sera were collected at 1 week after the final immunization; then, the mice were challenged with 5 LD_50_ of A/PR8 (H1N1) virus intranasally (i.n.) at 2 weeks after the final immunization. **(B–G)** Survival rates **(B,D,F)** and weight loss **(C,E,G)** were monitored for 14 days post-infection.

To investigate the protective efficacy of tetrameric NA vs. monomeric NA, the mice were immunized with NA tetramers or monomers as per the schedule shown in Figure 2A. As shown in Figures 2B–G, all the mice in the PBS control group showed signs of influenza, such as huddling or ruffled fur, from 2 to 3 days and died after the challenge with A/PR8 (H1N1) virus. The median survival days in the control group was 7 days. Vaccination with either H1N1_*P*__*R*__8_NA_*tet*_ or H1N1_*P*__*R*__8_NA_*mono*_ significantly protected the mice against the lethal dose of the A/PR8 (H1N1) influenza challenge (Figures 2B,C). All the H1N1_*P*__*R*__8_NA_*tet*_-immunized mice and 6/8 of H1N1_*P*__*R*__8_NA_*mono*_-immunized mice survived the influenza virus challenge (Figure 2B). Although the survival rates were not significantly different between the tetramer- and monomer-immunized mice, the mice immunized with H1N1_*P*__*R*__8_NA_*tet*_ showed significantly less weight loss compared with those immunized with H1N1_*P*__*R*__8_NA_*mono*_ (Figure 2C).

The cross-subtype protection was evaluated in mice that were immunized with H5N1 and H7N9 NA by heterosubtypic challenge with A/PR8 (H1N1) virus. As shown in Figures 2D,E, immunization with H5N1 NA tetramer significantly improved the survival of mice post-challenge with the A/PR8 (H1N1) virus. Then, 75% (6/8) of the mice that were immunized with H5N1_*V*__*N*_NA_*tet*_ tetramer survived, while all the mice in the control group died. However, the protection induced by H5N1 NA monomer is significantly weaker than that induced by tetramer (*p* < 0.05, log-rank test), with only 37.5% (3/8) of H5N1_*V*__*N*_NA_*mono*_-immunized mice surviving. No protection was observed in mice that were immunized with either H7N9_*S*__*H*_NA_*tet*_ or H7N9_*S*__*H*_NA_*mono*_ proteins (Figures 2E,F), indicating that protection induced by the NA protein is subtype-specific. Similar results were observed in tetrameric H1N1 and H3N2 NA proteins immunization. Tetrameric H1N1_*pdm09*_NA induced moderate cross-protection against A/PR8 (H1N1) virus infection while H3N2_*HK*_NA not (Supplementary Figure S2).

### Neuraminidase Tetramers Exhibited Better Abilities in Inducing Neuraminidase-Specific, Cross-Binding, and Neuraminidase Inhibition Antibodies That Were Related to Protection

**FIGURE 3:**
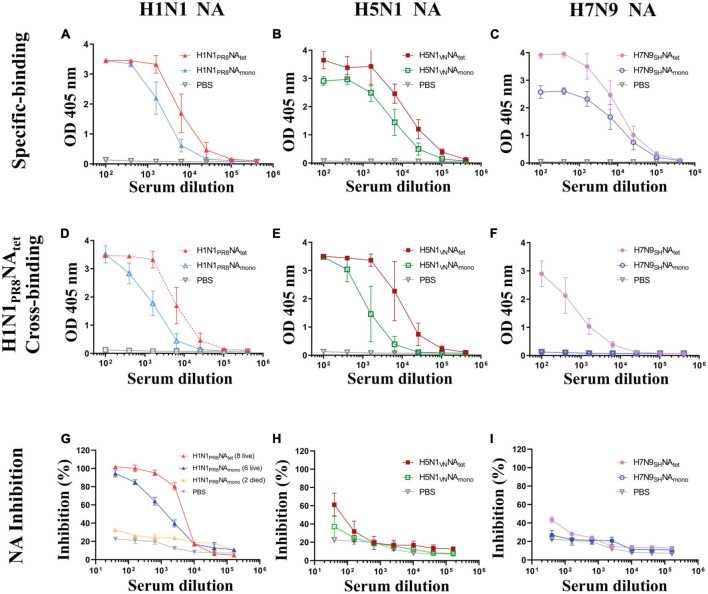
Humoral response induced tetrameric and monomeric neuraminidase (NA) proteins. The experimental design for immunization and challenge studies was identical to that detailed in the legend of Figure 2A. Serum were collected at 1 week after the final immunization. **(A–C)** The sera of mice vaccinated, respectively, with H1N1_*P*__*R*__8_NA_*tet*_ and H1N1_*P*__*R*__8_NA_*mono*_ **(A)**, H5N1_*V*__*N*_NA_*tet*_ and H5N1_*V*__*N*_NA_*mono*_ **(B)**, and H7N9_*S*__*H*_NA_*tet*_ and H7N9_*S*__*H*_NA_*mono*_ **(C)** proteins were tested for NA-specific binding antibody levels to the respective immunogen *via* ELISA. **(D–F)** The same sera whose results are shown in panels **(A–C)** were tested for cross-binding activity to H1N1_*P*__*R*__8_NA_*tet*_ protein *via* ELISA, respectively. **(G–I)** The same sera mentioned above were tested for NA inhibition (NAI) activity against NA of A/PR8 (H1N1) *via* enzyme-linked lectin assay (ELLA), respectively.

To explore the immunogenicity of tetrameric and monomeric NA proteins, the sera samples which were collected 1 week after the final immunization (Figure 2A) were tested for NA-reactive antibodies, including NA-specific binding antibodies to the respective immunogen, cross-subtype binding antibodies against NA of A/PR8 (H1N1), and NAI antibodies. The subtype of NA-specific antibodies was also analyzed. We firstly evaluated NA-specific binding antibodies against their respective immunogens by ELISA. As shown in Figure 3A, both of the H1N1 NA tetramer and monomer induced high levels of binding antibodies to the immunogens after two immunizations in mice. The H1N1 NA tetramer induced higher binding antibodies compared to H1N1 NA monomers (1:25,600 vs. 1:6,400). Similar results were observed between the groups of H5N1_*V*__*N*_NA_*tet*_ and H5N1_*V*__*N*_NA_*mono*_ (Figure 3B) as well as groups of H7N9_*S*__*H*_NA_*tet*_ and H7N9_*S*__*H*_NA_*mono*_ (Figure 3C). These results suggested that the NA tetramer with natural conformation has higher immunogenicity than the monomer.

Then, we evaluated the cross-subtype binding antibodies against H1N1 PR8 NA induced by the H5N1 NA and H7N9 NA proteins. The cross-binding of sera from different mice groups against the H1N1_*P*__*R*__8_NA_*tet*_ protein was tested by ELISA. Both H1N1 PR8 NA and H5N1 NA induced binding antibodies to H1N1_*P*__*R*__8_NA_*tet*_ (Figures 3D,E). The antibody levels induced by NA tetramer were higher than those by the monomer as previously indicated. The sera from mice immunized with H7N9 NA tetramer weakly reacted with H1N1_*P*__*R*__8_NA_*tet*_, while the sera from mice immunized with H7N9 NA monomer did not react with H1N1_*P*__*R*__8_NA_*tet*_ (Figure 3F). These results indicated that cross-binding antibodies are related to protection, while antibodies induced by NA proteins were mainly subtype-specific.

We also investigated the subtypes of NA-specific antibodies by isotyping ELISA assays. All tetrameric and monomeric NA proteins mainly induced IgG1 isotype of NA-specific antibodies (Supplementary Figure S1), indicating that the NA protein elicited a Th2-directed immune response.

Since it has been reported that the NA-induced protection was correlated with NAI antibodies (Couch et al., 2013; Memoli et al., 2016; Gilchuk et al., 2019; Stadlbauer et al., 2019; Vogel and Manicassamy, 2020), we evaluated the NAI activity of sera by ELLA. As shown in Figure 3G, sera from eight mice immunized with the H1N1_*P*__*R*__8_NA_*tet*_ tetramer exhibited a higher NAI activity against the NA of A/PR8 (H1N1) than the six survivors that were immunized with H1N1_*P*__*R*__8_NA_*mono*_. In contrast, the NAI activity was barely detectable in sera from two deceased mice in this group (Figure 3G). The NAI activity against the NA of A/PR8 (H1N1) was only weakly detected in mice that were immunized with H5N1 NA tetramer and monomer (Figure 3H) and almost not detected in mice that were immunized with H7N9 NA proteins (Figure 3I), suggesting that NAI antibodies may be related to protection during influenza virus infection. Taken together, those results mentioned above indicated that NA_*tet*_ exhibited better ability in inducing NA- specific-, cross- binding-, and NAI antibodies. Furthermore, NA_*tet*_ proteins provide better homogeneous protection or cross-protection than NA_*mono*_.

### Adjuvant Effects on the Immunity Induced by Neuraminidase Proteins

**FIGURE 4:**
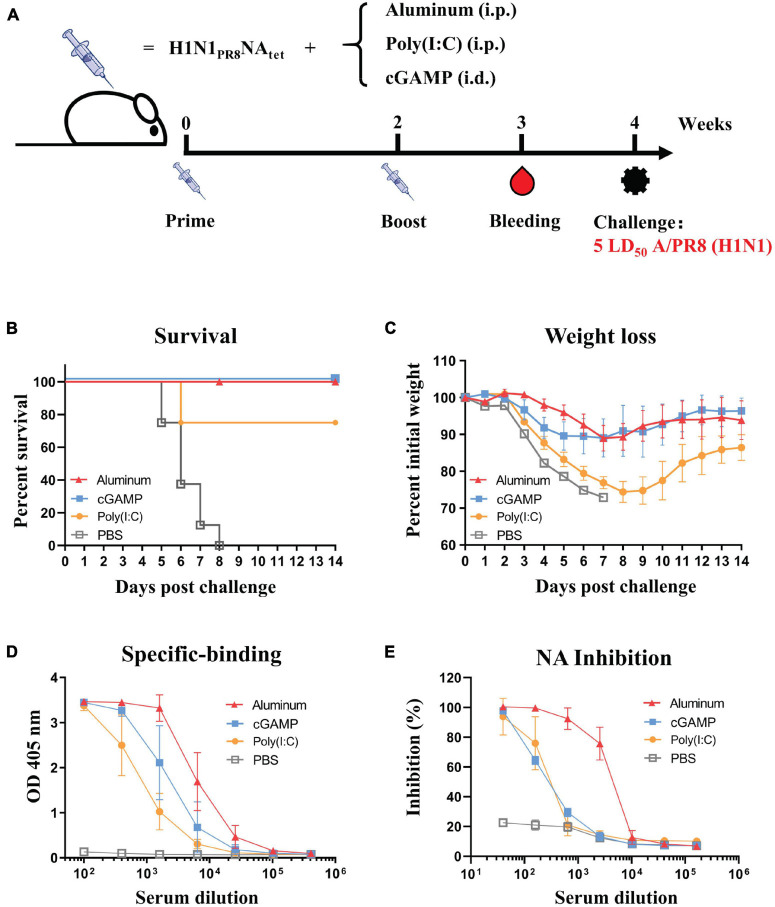
Effect of three adjuvants—aluminum, cGAMP, and Poly(I:C)—on the protective efficacy and humoral response induced by H1N1_*P*__*R*__8_NA_*tet*_ tetramer. **(A)** Six- to eight-week-old BALB/c mice were immunized twice at 2-week interval with 20 μg of H1N1_*P*__*R*__8_NA_*tet*_ protein adjuvanted with aluminum (*n* = 8, i.p.), cGAMP (*n* = 4, i.c.), or Poly(I:C) (*n* = 4, i.p.), respectively. The mice were immunized with phosphate-buffered saline (PBS) as control (*n* = 4, i.p.). Sera were collected at 1 week after the final immunization; then, the mice were challenged with 5 LD_50_ of A/PR8 (H1N1) virus intranasally (i.n.) at 2 weeks after the final immunization. **(B,C)** The survival rates **(B)** and weight loss **(C)** were monitored for 14 days post-infection. **(D)** The serum from individual mice in each experimental group was tested for neuraminidase (NA)-specific antibody levels against H1N1_*P*__*R*__8_NA_*tet*_ protein *via* ELISA. **(E)** The NAI activity of mice serum against NA of A/PR8 (H1N1) was tested *via* enzyme-linked lectin assay (ELLA).

We compared the effect of three adjuvants, aluminum, cGAMP, and Poly(I:C), on the protective efficacy and humoral responses induced by the H1N1_*P*__*R*__8_NA_*tet*_ tetramer. The immunization and challenge schedule is shown in Figure 4A. We found that complete protection against homologous A/PR8 (H1N1) virus challenge was observed in groups of aluminum or cGAMP (Figures 4B,C). The protection efficacy in the Poly(I:C) group was lower than those in the aluminum and cGAMP groups, as indicated by both the lower survival rates and weight loss (Figures 4B,C). We then evaluated the antibody responses in mice with different adjuvants of groups. Aluminum-adjuvant H1N1PR8NAtet induced the highest NA-specific binding antibodies and NAI antibodies (Figures 4D,E). cGAMP-adjuvant H1N1_*P*__*R*__8_NA_*tet*_ induced higher NA-specific binding antibodies than Poly(I:C)-adjuvant, but both adjuvants induced a similar level of NAI antibodies (Figures 4D,E). These results together suggested that tetrameric NA protein with natural conformation adjuvanted with aluminum elicited better protection than with cGAMP or Poly(I:C).

## Discussion

Influenza NA protein has been considered a potential target to develop universal vaccines that can provide cross-protection against different subtypes of influenza virus. The current commercial influenza vaccines are not efficient at inducing NA-specific protective immune response (Wohlbold et al., 2015; Chen et al., 2018; McMahon et al., 2020). Several NA-based vaccines, including recombinant NA proteins (Martinet et al., 1997; Bosch et al., 2010; Subathra et al., 2014; Liu et al., 2015; Wohlbold et al., 2015), DNA vaccines (Sandbulte et al., 2007), and virus-like particles (VLP) vaccines (Quan et al., 2012; Smith et al., 2017; Kim et al., 2019), have been evaluated in experimental animals and successfully provoked protection against influenza viruses. However, the mechanism of NA-induced protection has not been completely understood. Here we evaluated the immune response and protective efficacy of recombinant NA proteins. We found that the NA protein is highly immunogenic and induced protection against influenza viruses. Compared to the NA monomer, the NA tetramer is more immunogenic to induce higher NA-specific and cross-reactive biding antibodies, which are related to protection. The NA-reactive antibodies that bound to the native NA tetramers of the live virus might also play an important a role in inhibiting the viral release and spread of the infection. However, the protection is NA-subtype specific. H7N9_*S*__*H*_NA_*tet*_ tetramer could not provide heterosubtypic protection against A/PR8 (H1N1) virus infection. This finding is supported by the report that vaccination with the recombinant NA protein of A/PR8 (H1N1) could provide complete homologous protection against A/PR8 (H1N1) virus infection but not heterosubtypic protection against H3N2 virus (Wohlbold et al., 2015). Since there are 11 known NA subtypes, further studies may be required to explore an immune strategy, such as sequential or mixed immunization, to induce a broad immune response against all the 11 NA subtypes.

It was suggested that the protection induced by the NA protein was mediated by NAI antibodies (Couch et al., 2013; Memoli et al., 2016; Gilchuk et al., 2019; Stadlbauer et al., 2019; Vogel and Manicassamy, 2020). In this paper, we found that the protection induced by the NA protein was correlated with NAI antibodies. High NAI antibodies were detected in sera from the mice that survived the challenge, while NAI antibodies were undetectable in deceased mice. We also observed that NAI antibodies were strain-specific, while H5N1_*V*__*N*_NA_*tet*_ induced cross-protection against A/PR8 (H1N1) virus infection without provoking cross-reactive NAI antibodies, which probably depended on the differences among the epitopes around the active enzyme sites (Liu et al., 2015). However, H5N1_*V*__*N*_NA_*tet*_ induced high titers of subtype-specific binding antibodies to the NA protein of A/PR8 (H1N1) virus. Those cross-binding antibodies may potentially contribute to partial cross-protection. Further study is required to clarify whether the cross-protection was correlated with the NA-binding antibodies.

In addition, adjuvant is also an important consideration in NA protein-based vaccine design since we found that it worked on the immunity induced by the NA proteins. In contrast, we found that the mice of aluminum and cGAMP groups all survived the lethal influenza virus challenge, although the NAI antibody level of the aluminum group was slightly higher. We speculate that it may refer to the property of adjuvants or immune methods (i.p. vs. i.d.), but this still remains to be further determined. In short, adjuvant aluminum might be preferred for NA protein-based vaccination.

## Conclusion

In conclusion, our data suggests that tetrameric NA provides better homologous protection against influenza virus infection, and it could also confer preferable subtype-specific cross-protection. NA-reactive binding and inhibition antibodies are related to protection. Furthermore, aluminum adjuvant is preferential in vaccination of recombinant NA protein than cGAMP and Poly(I:C). We hope that this information could be useful for influenza vaccine formulation and administration.

## Data Availability Statement

The original contributions presented in the study are included in the article/Supplementary Material, further inquiries can be directed to the corresponding authors.

## Ethics Statement

The animal study was reviewed and approved by the Ethics Committee of Shanghai Public Health Clinical Center.

## Author Contributions

JH conceived and designed the experiments and supervised the project. XD, ML, and QZ expressed and purified the recombinant NA proteins. XD performed the MUNANA-based enzymatic activity assay and ELISA and ELLA assays. XD and QW performed the mice experiments. XD, JH, and FW analyzed the data and wrote the manuscript. All authors have read and agreed to the published version of the manuscript.

## Conflict of Interest

The authors declare that the research was conducted in the absence of any commercial or financial relationships that could be construed as a potential conflict of interest.

## Publisher’s Note

All claims expressed in this article are solely those of the authors and do not necessarily represent those of their affiliated organizations, or those of the publisher, the editors and the reviewers. Any product that may be evaluated in this article, or claim that may be made by its manufacturer, is not guaranteed or endorsed by the publisher.
