# Supplementary material for: Tetrameric Neuraminidase of Influenza A Virus Is Required to Induce Protective Antibody Responses in Mice
Source: Front Microbiol. 2021 Oct 4;12:729914. doi: 10.3389/fmicb.2021.729914 (PMC8521146; doi:10.3389/fmicb.2021.729914)
Supplement: Supplementary file 1 [file Data_Sheet_1.pdf]

Supplementary Figures:

Figure S1. Antibody isotyping of mice serum vaccinated with diverse subtypes of NAtet or NAmono proteins. (A to F) The isotypes of NA-specific antibodies of serum from mice vaccinated with $H1N1_{PR8}NA_{tet}$ (A), $H1N1_{PR8}NA_{mono}$ (B), $H5N1_{VN}NA_{tet}$ (C), $H5N1_{VN}NA_{mono}$ (D), $H7N9_{SH}NA_{tet}$ (F) and $H7N9_{SH}NA_{mono}$ (G) were determined via ELISA.

Figure S2. The protective efficacy of $H1N1_{p09}NA_{tet}$ and $H3N2_{HK}NA_{tet}$. (A) The experimental design for immunization and challenge studies. Six- to eight-week-old BALB/c mice were immunized twice at 2-week interval with 20 μg of $H1N1_{p09}NA_{tet}$ and $H3N2_{HK}NA_{tet}$ proteins adjuvanted with aluminum (i.p.) respectively. Mice were challenged with 5 LD_{50} of A/PR8 (H1N1) virus intranasally (i.n.) two weeks after the final immunization. (B to E) Survival rates (B and D) and weight loss (C and E) were monitored for 14 days post-infection.

Figure S1

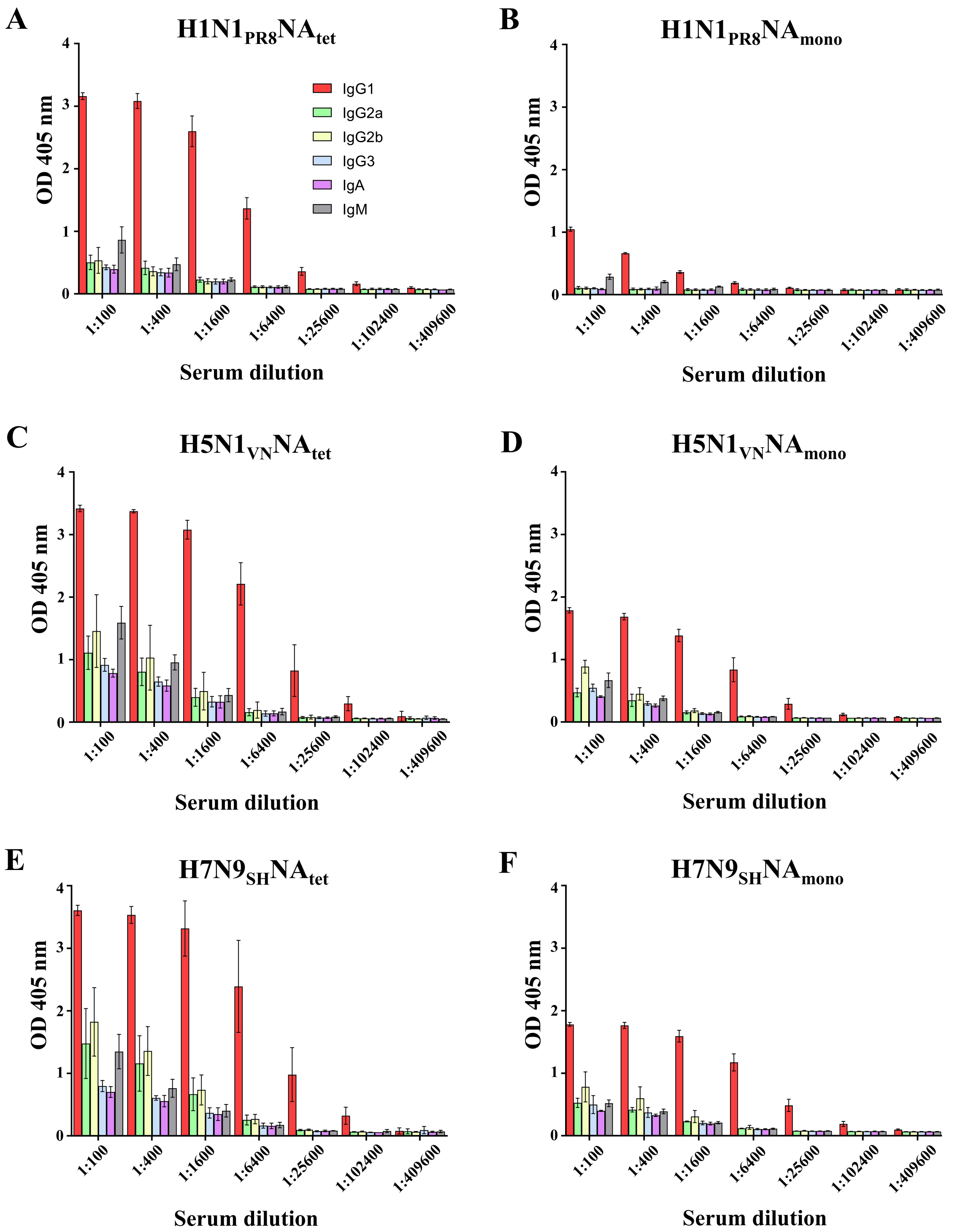
A
H1N1PR8NAtet
B
H1N1PR8NAmono
C
H5N1VNNAtet
D
H5N1VNNAmono
E
H7N9SHNAtet
F
H7N9SHNAmono
IgG1
IgG2a
IgG2b
IgG3
IgA
IgM
OD 405 nm
Serum dilution
1:100
1:400
1:1600
1:6400
1:25600
1:102400
1:409600
0
1
2
3
4

Figure S2

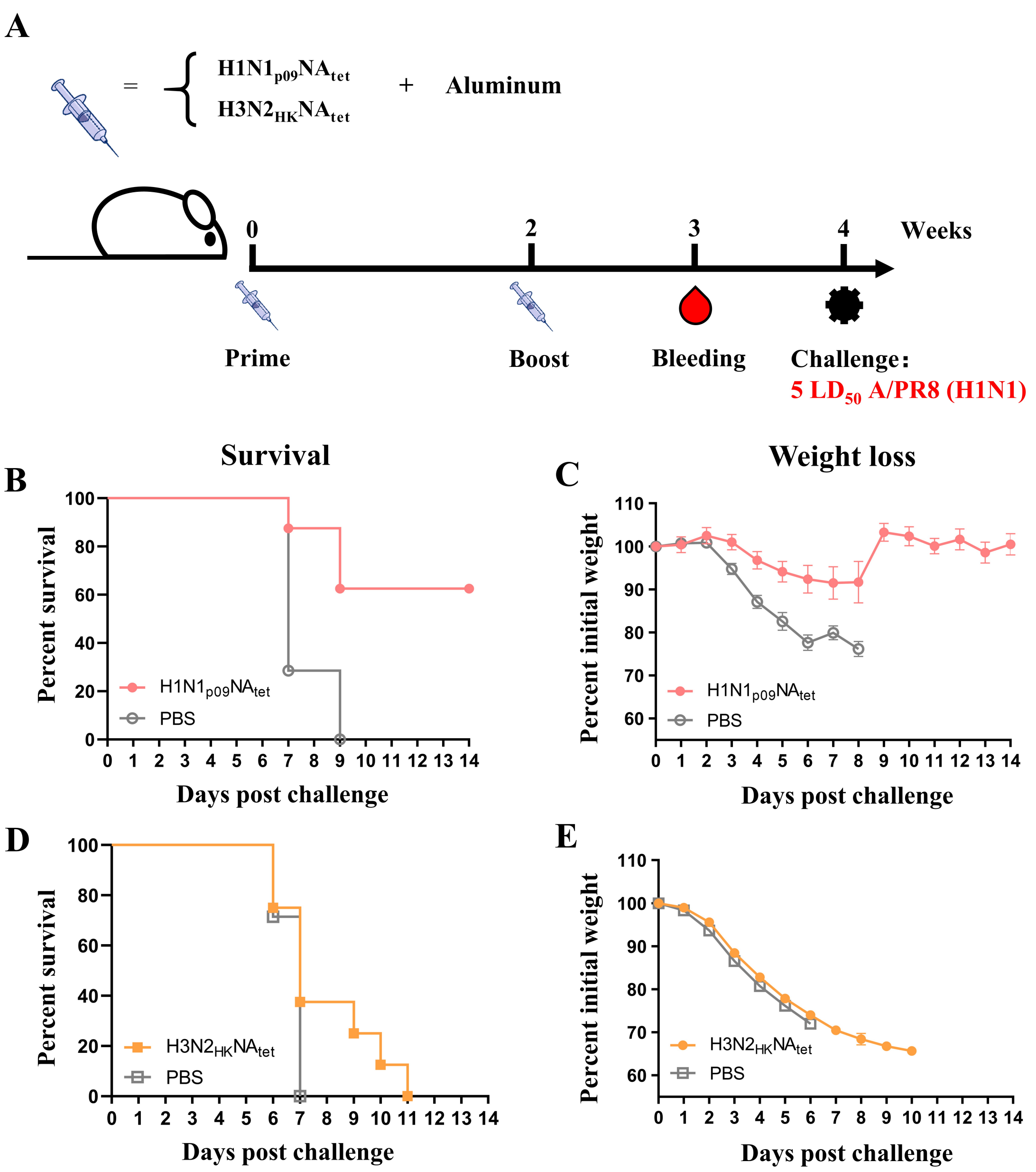

A
H1N1p09NAtet
H3N2HKNAtet
+
Aluminum
0
2
3
4
Weeks
Prime
Boost
Bleeding
Challenge:
5 LD50 A/PR8 (H1N1)
Survival
B
Percent survival
H1N1p09NAtet
PBS
Days post challenge
Weight loss
C
Percent initial weight
H1N1p09NAtet
PBS
Days post challenge
D
Percent survival
H3N2HKNAtet
PBS
Days post challenge
E
Percent initial weight
H3N2HKNAtet
PBS
Days post challenge
